# Oridonin attenuates diclofenac-induced organ damage by suppressing endoplasmic reticulum stress and inflammasome activation

**DOI:** 10.3389/ftox.2026.1821778

**Published:** 2026-05-22

**Authors:** Ali H. Al-Baldawi, Mahmoud M. Samaha, Marwa S. Zaghloul

**Affiliations:** Department of Pharmacology and Toxicology, Faculty of Pharmacy, Mansoura University, Mansoura, Egypt

**Keywords:** diclofenac, drug-induced organ toxicity, endoplasmic reticulum stress, inflammasomes, oxidative stress

## Abstract

**Background:**

Diclofenac is a non-steroidal anti-inflammatory drug (NSAID) extensively prescribed in clinical practice; its use can be associated with toxic effects on multiple organs. These deleterious effects are associated with a combination of oxidative stress, sterile inflammation, and disrupted epithelial barrier integrity. Oridonin has strong antioxidant and anti-inflammatory characteristics, making it a potential candidate for NSAID-induced organ damage.

**Objective:**

Evaluate the protective effects of oridonin against diclofenac-induced liver, kidney and stomach toxicity, while elucidating molecular mechanisms involved in oxidative stress modulation, endoplasmic reticulum (ER) stress suppression, inflammasome inhibition and barrier preservation of epithelial cells.

**Methods:**

30 male Sprague-Dawley rats were divided into five groups: normal control, oridonin control, diclofenac (100 mg/kg, IP), and diclofenac treated with low-dose (10 mg/kg) or high-dose (20 mg/kg) oridonin were the experimental conditions. Renal and hepatic function was assessed by serum and urinary markers. Oxidative stress was assessed by the determination of MDA, GSH, and total antioxidant capacity (TAC) in tissue homogenates. Molecular pathways were studied by ELISA and Western blotting to quantify ER stress markers (IRE1, CHOP, PERK) and the TXNIP/NLRP3/Caspase-1 inflammasome axis. Gastric barrier integrity was confirmed by expression measurement of tight junction proteins (Claudin, ZO-1, Occludin), histopathological, and immunohistochemical markers of NF-κB and IL-1β.

**Results:**

Oridonin pretreatment led to the recovery of antioxidant defenses and large decreases in the activation of the TXNIP/NLRP3/Caspase-1 axis, and ER stress signaling. In addition, oridonin maintained the gastric mucosal architecture and the expression of critical tight junction proteins and inhibited NF-κB-induced inflammation.

**Conclusion:**

Oridonin ameliorates diclofenac-induced multi-organ toxicity by reducing oxidative stress and ER stress, inhibiting the NLRP3 inflammasome, and preserving epithelial barrier integrity. Thus, oridonin can be considered a suitable drug to ameliorate the unwanted side effects of NSAID treatment.

## 1 Introduction

Diclofenac has been one of the most widely prescribed non-steroidal anti-inflammatory drugs (NSAIDs) internationally and acts as a mainstay in the treatment of pain and inflammatory diseases like rheumatoid arthritis and osteoarthritis. In terms of clinical application, its enormous global consumption (approximately 1,443 tons annually) is regarded as its position as the most widely consumed NSAID worldwide (Hernández-Zamora et al., 2025). Recent updates suggested that the medical use of diclofenac consistently produces various serious risks to health, particularly nephrotoxicity, hepatotoxicity, and gastric mucosal injury (Khatlan et al., 2024; Kunitsu et al., 2025).

According to epidemiological data, NSAIDs consumption correlates with 50%–70% increased risk of acute kidney injury (AKI), while diclofenac has been specifically noted to have an eight-fold higher susceptibility for kidney complications relative to the other agents (Khatlan et al., 2024; Kunitsu et al., 2025). Moreover, diclofenac is also one of the most common causes of idiosyncratic drug-induced liver injury (DILI) and the second most frequent agent in the population-based studies of DILI (Björnsson, 2016). From the perspective of gastrointestinal safety, NSAIDs are responsible for 15%–35% of all peptic ulcer complications, and the point prevalence of ulcer development is often up to 30% in chronic users (Singh and Triadafilopoulos, 1999). These adverse effects pose a significant global health challenge, and protective strategies need to be developed.

Diclofenac-induced multi-organ toxicities can be attributed to a complex interplay between oxidative stress and sterile inflammation, and this can be effectively monitored using specific biochemical and molecular biomarkers. Metabolic activation of the drug causes accumulation of reactive oxygen species (ROS) that result in lipid peroxidation, as shown by increased malondialdehyde (MDA) and diminished reduced glutathione (GSH) levels (Alabi and Akomolafe, 2020; Alkuraishy et al., 2019). This oxidative imbalance serves as a crucial upstream signal that triggers the activation of the TXNIP/NLRP3 inflammasome axis, further stimulating downstream caspase-1 signaling that is involved in driving pyroptotic cell death (Luo et al., 2024; Sbai et al., 2024). This inflammatory cascade precipitates organ dysfunction, characterized by high increases in serum transaminases (ALT, AST), urea, and creatinine (Alabi and Akomolafe, 2020; Khalil et al., 2025), and damages epithelial barrier integrity, with downregulation of important tight junction proteins like claudins and Zonula Occludens-1 (ZO-1) (Chao et al., 2021; Yanaka et al., 2007).

Diclofenac nephrotoxicity is often evident as AKI, with varying degrees of disturbance ranging from mild dysfunction to full renal failure (Alabi and Akomolafe, 2020). Mechanisms include reduced prostaglandin production via cyclooxygenase (COX) blockade, mitochondrial oxidative injury, and stimulation of inflammatory cascades; this results in significant loss of glomerular filtration and tubular necrosis (Pavani et al., 2022; Zhuang et al., 2018). Diclofenac causes hepatotoxicity mainly through mechanisms that disrupt prostaglandin and mitochondrial functions, cyclic ROS generation, impaired autophagy, inflammation, and pro-apoptotic actions, leading to hepatocyte apoptosis (Gómez-Lechón et al., 2003; Ramachandran et al., 2018). NSAID-induced gastric mucosal damage is mediated by prostaglandin inhibition, which results in prostaglandin blockade and direct loss of epithelial and mitochondrial cell membranes, culminating in ulceration, bleeding, and failure of epithelial barrier function (Chao et al., 2021; Lee et al., 2005; Yanaka et al., 2007). Diclofenac specifically inhibits tight junction (TJ) protein expression, including Claudin-1, Claudin-4, Claudin-18, Occludin, and ZO-1 (Chao et al., 2021; Lee et al., 2005; Mima et al., 2005; Yanaka et al., 2007). These proteins are essential for maintaining the TJ site and the gastric epithelial barrier. Their disturbance enhances the permeability of the gastric mucosa, inducing inflammation, leukocyte infiltration, and ultimately epithelial cell apoptosis, thereby reducing barrier function (Peng et al., 2023; Poritz et al., 2011; Wang et al., 2022).

Oridonin, a tetracyclic diterpenoid in Isodon rubescens (*Rabdosia rubescens*), is a key compound in traditional Chinese medicine (Li et al., 2021). Pharmacological studies in contemporary literature also demonstrate the therapeutic efficacy of oridonin in a variety of pathological conditions, including oncological, inflammatory, and autoimmune pathologies, as well as organ-specific toxicity (Bao et al., 2014; Xu et al., 2018). Oridonin’s pleiotropic effects are primarily attributable to its regulatory influence on redox homeostasis, cellular proliferation, endoplasmic reticulum (ER) stress responses, apoptotic pathways, and immune modulation mechanisms (Li et al., 2011; Zhao et al., 2023). Oridonin was chosen for this study due to its specific therapeutic profile that directly addresses the mechanism mediated by diclofenac toxicity (Li et al., 2021; Xu et al., 2018). Diclofenac promotes damage to organs via oxidative stress, NLRP3 inflammasome activation, and ER stress. Oridonin has been shown to act on the exact pathways to drive cytoprotection (Li et al., 2011; Zhao et al., 2023). Oridonin has been shown to activate the Nrf2 signaling pathway in earlier reports, through the neutralization of ROS (Chen et al., 2022; Yang et al., 2019) and acting as a specific covalent inhibitor of NLRP3-inflammasome with downstream inhibition of the production of pro-inflammatory cytokines like IL-1β (He et al., 2018; Ou et al., 2025). Additionally, oridonin has been reported to modulate ER stress and to maintain epithelial barrier intactness by upstream generation of TJ proteins, indicating that it may be specially adapted to attenuate the multi-mechanistic risk of anti-inflammatory actions elicited by NSAIDs (Li et al., 2018; Li et al., 2024; Zhu et al., 2025). Therefore, this study selected oridonin as a potential candidate to investigate its protective effects against diclofenac-induced multi-organ toxicity, through studying various molecular biomarkers.

## 2 Materials and methods

### 2.1 Animals

A total of 30 male Sprague-Dawley rats, weighing 230 ± 30 g and approximately 8 weeks old, were obtained from the Holding Company for Biological Products and Vaccines (VACSERA), Cairo, Giza, Egypt. Before use, the rats were kept under normal environmental conditions and allowed a 2-week acclimatization period in a standard laboratory environment within large, comfortable cages at the animal house of the Faculty of Pharmacy, Mansoura University. Ethical approval for the study was granted by the Mansoura University Animal Care and Use Committee and the Faculty of Pharmacy Research Ethics Committee, with approval code: MU-ACUC (PHARM.MS.24.11.121). The experiment was conducted according to the National Institutes of Health (NIH) guidelines, the Care and Use of Laboratory Animals (Publication No. 85–23, revised 1985), and the AVMA 2020 (Underwood and Anthony, 2020). The authors complied with the ARRIVE 2.0 guidelines (Percie du Sert et al., 2020).

### 2.2 Drugs and chemicals

Oridonin was purchased from Nanjing Yuanzhi Biotechnology Co., Ltd. (Jiangsu Province, China) and dispersed in 0.5% w/v sodium carboxymethylcellulose solution (CMC-Na) for oral administration. Diclofenac sodium was obtained as Voltaren® ampoules for injection from Novartis (Switzerland).

### 2.3 Experimental design

Five groups of rats (n = 6 per group) were randomly assigned in a complete block design: the normal control group received only 0.5% CMC-Na solution for 7 days; the oridonin control group received only 20 mg/kg of oridonin for 7 days by gastric gavage (Liu et al., 2019). The diclofenac group received 0.5% CMC-Na solution for 7 days, followed by a single intraperitoneal (IP) injection of 100 mg/kg diclofenac sodium on day 8 (Elnashar et al., 2024; Goswami et al., 2017). The oridonin 10 mg/kg group received 10 mg/kg oridonin for 7 days by gastric gavage (Li et al., 2022). The oridonin 20 mg/kg group received 20 mg/kg of oridonin by gastric gavage for 7 days. After 24 h (at day 8), rats in both previous groups received the same induction by diclofenac (Zang et al., 2016).

Animal welfare was carefully monitored on a daily basis throughout the study to prevent, minimize, and alleviate any potential pain or distress. Rats were observed daily for changes indicative of discomfort or distress, including alterations in general appearance (e.g., posture and grooming), behavior (e.g., activity level and vocalization), body weight, food and water consumption, and clinical signs such as respiratory rate and mobility. All assessments were conducted by trained personnel experienced in normal species-specific behavior and appearance. Animals were excluded from data analysis if they exhibited signs of unexpected illness, such as infection, or if they failed to receive the complete dose of diclofenac or oridonin due to technical issues during drug administration, anesthesia, or sample collection.

### 2.4 Sample collection

On day 8 and with water deprivation, urine was collected through individual metabolic cages (to separate urine from feces and food debris). Animals were placed in isolation during the collection period. Urine samples were examined for contamination; any that were deemed tainted were discarded. The aliquots were kept at very precise temperatures until determination (Alresheedi et al., 2025; Tokaryk, 2019).

Rats were closely monitored for any signs of stress or discomfort during confinement in metabolic cages including changes in activity level, respiration rate, and vocalization. If any animal exhibited persistent distress (e.g., agitation, vocalization, or self-injury), confinement was immediately terminated, and the animal was allowed to recover in its home cage. Monitoring was performed by trained personnel at 2 h intervals.

On day 9, the animals were anesthetized with thiopental sodium (40 mg/kg, IP) (Alresheedi et al., 2025). Capillary tubes were used to collect blood samples by means of the retro-orbital venous plexus. The blood was left to clot at room temperature, then centrifuged at 4,000 rpm for 15 min to obtain clear serum. Separated serum was stored in aliquots at −80 °C until required for subsequent biochemical assessments.

Additionally, the liver, stomach, and kidneys were harvested and cleaned with ice-cold saline. The left lobes of the liver, a part of the stomach body, and the left kidneys were obtained for histological analysis and immunostaining by fixation in 10% formalin-buffered saline. The large right lobes of the liver, the remaining part of the stomach body, and the right kidneys were homogenized in ice-cold phosphate-buffered saline (PBS) using an Omni tissue homogenizer (Omni TH homogenizer, France) to yield 10% (w/v) tissue homogenate. The homogenates were centrifuged at 4,000 rpm at 4 °C using a cooling centrifuge (Sigma D-37520, Sigma Laborzentrifugen GmbH, Germany). The supernatant was collected and stored at −80 °C until analysis.

Rats were followed 2-step euthanasia process; first rats were anesthetized with thiopental sodium (40 mg/kg, IP) for blood collection followed by exsanguination via cardiac puncture.

### 2.5 Assessment of liver and kidney functions

Aspartate aminotransferase (AST) (Cat# 11408005, LiquiCHEK™ AST) and alanine aminotransferase (ALT) activities in serum were measured using reagent kits (Cat# 11409005, LiquiCHEK™ ALT) obtained from Agappe Diagnostics Limited, Pattimattom, Ernakulam, Kerala, India. The analysis is based on an International Federation of Clinical Chemistry and Laboratory Medicine (IFCC)-recommended kinetic enzymatic method, measured spectrophotometrically (Thefeld et al., 1974).

Serum and urinary creatinine (Cat# MD1001111, Creatinine-J) were measured using reagent kits obtained from SPINREACT, S.A., Sant Esteve de Bas (Girona), Spain. The assay is based on the Jaffé reaction, a colorimetric kinetic method for creatinine determination (Kaplan and Glucose, 1984). Serum and urinary urea (Cat# TK41041, UREA-LQ) were measured using reagent kits obtained from SPINREACT, S.A., Sant Esteve de Bas (Girona), Spain. The assay is based on an enzymatic UV kinetic method using Urease and Glutamate Dehydrogenase (GLDH) (Kaplan and Glucose, 1984). The absorbance was read using a spectrophotometer (Labomed, Los Angeles, USA)

### 2.6 Assessment of oxidant/antioxidant parameters in tissue homogenates

Malondialdehyde (MDA) (Cat# MD2529), reduced glutathione (GSH) (Cat# GR2511), and total antioxidant capacity (TAC) (Cat# TA2513) levels in liver, stomach, and kidney homogenates were determined using commercially available Bio-Diagnostic colorimetric assay kits (Giza, Egypt), in accordance with the manufacturer’s instructions. Absorbance was measured using a spectrophotometer (Labomed, Los Angeles, USA).

### 2.7 Enzyme-linked immunosorbent assay (ELISA)

Using commercially available rat ELISA kits, the hepatic and renal expressions of inositol-requiring enzyme 1 (IRE1) (Cat# MBS2503494; MyBioSource, Inc.; San Diego, California, United States), thioredoxin-interacting protein (TXNIP) (Cat# ELK0998; ELK Biotechnology Co., Ltd., Wuhan, Hubei, China), and caspase-1 (Cat# E-EL-R0371; Elabscience Biotechnology Co., Ltd., Wuhan, Hubei, China) were assessed.

For gastric analysis, the expression of tight junction proteins was evaluated using ELISA kits specific for Zonula Occludens-1 (ZO-1) (Cat# MBS706128; MyBioSource, Inc.; San Diego, California, United States) and Claudin-1 (CLDN1) (Cat# MBS731496; MyBioSource, Inc.; San Diego, California, United States). The used microplate reader (Bio Tek Instruments ELx800, Winooski, VT, USA) was set to λ = 450 nm.

### 2.8 Western blotting analysis

The protein expression levels of C/EBP Homologous Protein (CHOP), Protein Kinase RNA-like Endoplasmic Reticulum Kinase (PERK), and NOD-like Receptor Family Pyrin Domain-Containing 3 (NLRP3) were evaluated in liver and kidney tissues using Western blot analysis. Tissue samples were homogenized in RIPA lysis buffer (Bio Basic, Cat# PL005) supplemented with protease and phosphatase inhibitor cocktails, incubated on ice for 30 min, and centrifuged at approximately 16,000 × g for 30 min at 4 °C. The resulting supernatants were collected, and total protein concentrations were determined using the Bradford assay (Bio Basic, Cat# SK3041).

Equal amounts of protein (20 µg) were mixed with an equal volume of 2× Laemmli sample buffer (4% SDS, 10% 2-mercaptoethanol, 20% glycerol, 0.004% bromophenol blue, 0.125 M Tris-HCl, pH 6.8), boiled for 5 min, and separated by SDS-PAGE using TGX Stain-Free™ FastCast™ gels (Bio-Rad, Cat# 161-0181). Electrophoresis was performed at 50 V for 20 min during the stacking phase, followed by 100–150 V until adequate protein separation was achieved. Proteins were then transferred onto PVDF membranes using a Trans-Blot Turbo system at 25 V for 7 min. Transfer efficiency was verified using stain-free blot imaging on a ChemiDoc™ imaging system.

Membranes were blocked for 1 h at room temperature in TBST containing 3% bovine serum albumin (20 mM Tris-HCl, pH 7.5; 150 mM NaCl; 0.1% Tween-20), followed by overnight incubation at 4 °C with the appropriate primary antibodies diluted in blocking buffer. The primary antibodies used were rabbit polyclonal anti-CHOP/DDIT3 (Thermo Fisher Scientific, Cat# PA5-102305; 1:500–1:2,000), rabbit polyclonal anti-PERK/EIF2AK3 (Thermo Fisher Scientific, Cat# PA5-99447; 1:500–1:2,000), and rabbit polyclonal anti-NLRP3 (Thermo Fisher Scientific, Cat# PA5-79740; 0.1–0.5 μg/mL). For loading control normalization, membranes were probed with mouse monoclonal anti-β-actin (Thermo Fisher Scientific, Cat# MA1-140; 1:5,000–1:20,000).

Following primary antibody incubation, membranes were washed three to five times with TBST (5 min each) and then incubated for 1 h at room temperature with the appropriate horseradish peroxidase-conjugated secondary antibodies. After additional washing, immunoreactive bands were visualized using Clarity™ Western ECL substrate (Bio-Rad, Cat# 170-5060). Chemiluminescent signals were captured using a ChemiDoc™ imaging system, and band intensities were quantified using image analysis software. Target protein expression levels were normalized to β-actin and expressed relative to control samples.

### 2.9 Histopathological and immunohistochemical examination

Tissue samples fixed in formalin were dehydrated through graded ethanol concentrations, cleared in xylene, and embedded in paraffin wax. The paraffin blocks were cut at a thickness of 5 µm using a microtome. The sections obtained were then stained with hematoxylin and eosin (H&E) and viewed under a light microscope (Olympus CH2, Japan). Histopathological examination was performed in a blinded manner, with all slides coded prior to examination. For each animal, four sections per slide were evaluated and the average of each 4 scores was calculated and used for statistical analysis and scattered dote graph presentation.

Gastric mucosal injury was evaluated according to a modified 4-point histopathological scoring system (Sun et al., 2018). Microscopically, edema, erosion, ulceration, and necrosis was assessed, and the presence of each pathological feature was assigned a score of 1, with the absence of damage assigned a score of 0. The cumulative histopathological score of each specimen was obtained by summing the individual parameters. Six randomly selected microscopic fields were studied and assessed for each experimental group.

Hepatic tissue damage was analyzed by a semi-quantitative grading system ranging from 0 to 4. In brief, score 0 indicated normal hepatic architecture; score 1 denoted minimal lesions, score 2 mild lesions, score 3 moderate lesions, and score 4 severe hepatic damage (Malý et al., 2019).

Assessment of renal histopathology was confined to renal cortical sections to facilitate assessment of tubular and glomerular injury scoring. Tubular injury was characterized by tubular dilation, tubular necrosis, hydropic degeneration, and tubular epithelial cell sloughing. The severity of injury was based on the following: score 0, no tubular injury; score 1, <10% of tubules affected; score 2, 10%–25% affected; score 3, 26%–50% affected; score 4, 51%–75% affected; score 5, >75% of tubules affected (Toprak et al., 2020). Glomerular injury was assessed using a semi-quantitative scoring system ranging from 0 to 3, where score 0 indicated normal glomerular structure; score 1 represented thickening of Bowman’s capsule; score 2 denoted retraction of the glomerular tuft; and score 3 indicated glomerular fibrosis (De Miguel et al., 2019).

The expression levels of nuclear factor kappa-light-chain-enhancer of activated B cells (NF-κB) and interleukin-1β (IL-1β) in hepatic and renal tissues and occluding in stomach tissues were assessed by immunostaining using the Avidin-Biotin Complex method (Hsu and Raine, 1981). Tissue sections were deparaffinized and rehydrated, then subjected to high-pressure antigen retrieval in 10 mM citrate buffer (pH 6.0) as recommended for the primary antibodies.

The sections were then incubated with the following primary antibodies: NF-κB: NF-κB p65/RelA Rabbit Polyclonal Antibody (Cat# A2547; ABclonal, Woburn, MA, USA). IL-1β: Anti-IL-1β Rabbit Polyclonal Antibody (Cat# GB11113; ServiceBio, Wuhan, China). Occludin: Anti-Occludin Rabbit Polyclonal Antibody (Cat# A2601; ABclonal, Woburn, MA, USA). The antibodies were used at 1:200 for NF-κB and 1:800 for IL-1β and occludin.

The stained sections were evaluated under a light microscope (Olympus CH2, Japan) in a blinded manner, with slides coded before examination to ensure unbiased assessment. Examination was recorded in 4 sections per slide (each slide representing one animal), and the average of the 4 results was calculated and used for statistical analysis.

Whole-tissue sections were processed for high-resolution images in ImageJ. A virtual dissection or extraction of the ROI (region of interest) in whole tissue sections at magnifications up to ×40 was performed to estimate relative antigen content. The digital images were processed using the color deconvolution method to separate the brown DAB chromogen from the hematoxylin counterstain. A monochrome image representing the DAB content was then subjected to frequency analysis using ImageJ Software (FIJI, National Institutes of Health, USA), and the area fraction of DAB (antigen) was calculated. The results were expressed as the percentage of immunopositively stained area in each hepatic, renal and stomach sections and presented as means ± SEM (Helps et al., 2012).

### 2.10 Statistical analysis

All analyses were conducted using GraphPad Prism version 10.6.1 for Windows (GraphPad Software, Boston, Massachusetts, USA; www.graphpad.com). Normality was assessed with the Shapiro-Wilk test. For normally distributed variables, a one-way ANOVA with Tukey’s multiple comparisons test was performed. For variables that did not follow a normal distribution, the Kruskal–Wallis test with all pairwise comparisons using Dunn’s method was applied. Data are presented as mean ± standard deviation (SD) for parametric data or median and interquartile range (IQR) for non-parametric scoring. A *P*-value of ≤0.05 was considered statistically significant.

## 3 Results

No statistically significant differences were observed in any of the assessed parameters in the oridonin control group versus the normal control group.

### 3.1 Influence of oridonin on kidney functions

**FIGURE 1:**
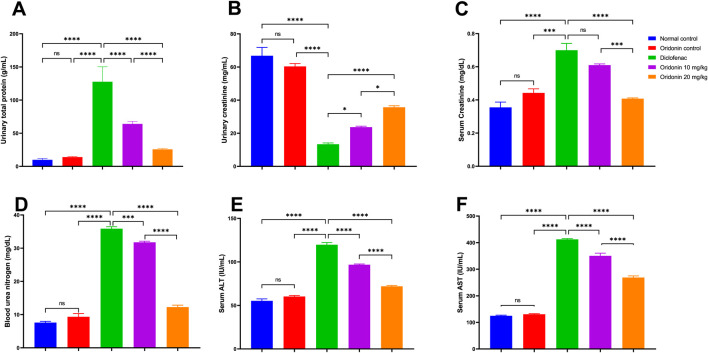
Influence of oridonin on the alterations in kidney and liver functions induced by diclofenac. **(A)** urinary total protein, **(B)** urinary creatinine, **(C)** serum creatinine, **(D)** blood urea nitrogen (BUN), **(E)** serum ALT, **(F)** serum AST. ALT: alanine aminotransferase, AST: Aspartate aminotransferase. Data are expressed as mean ± SD; n = 6. Rats received oridonin (10 mg/kg and 20 mg/kg) for 7 days by gastric gavage followed by diclofenac (100 mg/kg, single IP dose) on the 8th day; * denotes p-value <0.05, ** denotes p-value <0.01, *** denotes p-value <0.001, **** denotes p-value <0.0001; This was determined using one-way ANOVA followed by Tukey multiple comparison *post hoc* tests.

Administration of diclofenac markedly increased total urinary protein excretion by about 13 folds compared to normal control. Simultaneously, urinary creatinine decreased by nearly 80%. Serum creatinine and blood urea nitrogen (BUN) were significantly increased in the diclofenac group, by around 97% and 372%, respectively, in relation to the normal control (Figure 1).

Compared to the diclofenac group, renal dysfunction was significantly relieved with oridonin 10 mg/kg pretreatment. The excretion of total urinary protein was markedly decreased by 50%, and urinary creatinine was raised by 77%. BUN was significantly reduced by 11%, as compared with the diclofenac-treated rats. In a similar manner, oridonin 20 mg/kg induced more pronounced reno-protective effects compared to diclofenac treatment. Total urinary protein excretion was markedly decreased by ∼80%, and urinary creatinine increased by around 167%. Serum creatinine and BUN levels were reduced by approximately 42% and 66%, respectively, compared with diclofenac-treated rats (Figure 1).

A direct comparison of both oridonin doses showed that oridonin 20 mg/kg was more effective than oridonin 10 mg/kg, further decreasing total urinary protein excretion by about 59%, raising urinary creatinine by around 51%, and providing additional decreases in the serum creatinine and BUN levels of about 33% and 61%, respectively (Figure 1).

### 3.2 Influence of oridonin on liver functions

Serum ALT activity was increased by an estimated 117% with diclofenac compared to the normal control. In a similar manner, serum AST activity increased significantly by about 231% compared with the normal control (Figure 1).

Treatment with oridonin 10 mg/kg attenuated hepatic enzyme elevation compared to the diclofenac group. Serum ALT activity was affected by about 19% decrease, while serum AST activity decreased by around 15% decrease. Oridonin 20 mg/kg produced a more pronounced hepatoprotective reactions than diclofenac treatment. Serum ALT and AST activities were reduced by 40% and 35%, respectively (Figure 1).

The two doses of oridonin were directly compared, and the results indicated a significantly higher efficacy of oridonin 20 mg/kg as indicated by lowering serum ALT and AST activities by about 26% and 23%, respectively, over oridonin 10 mg/kg (Figure 1).

### 3.3 Influence of oridonin on oxidant/antioxidant parameters in tissue homogenates

**FIGURE 2:**
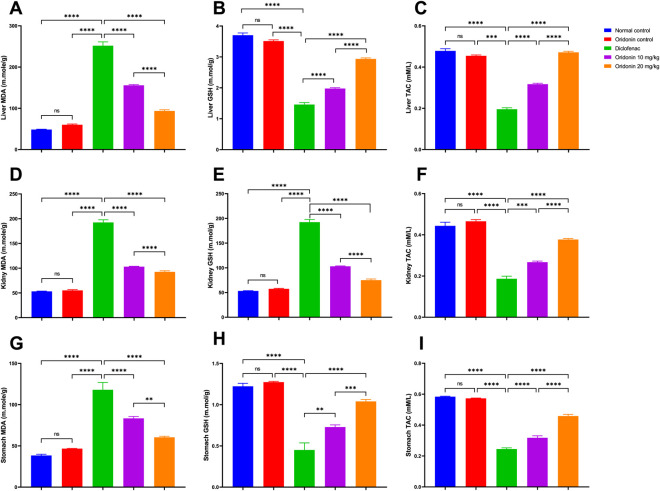
Influence of oridonin on the alterations in oxidant/antioxidant parameters induced by diclofenac in tissue homogenates. **(A)** Liver MDA, **(B)** Liver GSH, **(C)** Liver TAC, **(D)** Kidney MDA, **(E)** Kidney GSH, **(F)** Kidney TAC, **(G)** Stomach MDA, **(H)** Stomach GSH, **(I)** Stomach TAC. MDA: Malondialdehyde, GSH: reduced glutathione, TAC: total anti-oxidant capacity. Data are expressed as mean ± SD; n = 6. Rats received oridonin (10 mg/kg and 20 mg/kg) for 7 days by gastric gavage followed by diclofenac (100 mg/kg, single IP dose) on the 8th day; ** denotes p-value <0.01, *** denotes p-value <0.001, **** denotes p-value <0.0001; This was determined using one-way ANOVA followed by Tukey multiple comparison *post hoc* tests.

Comparison between the normal control group and the diclofenac group showed marked oxidative imbalance in all studied tissues. Compared with normal controls, diclofenac administration in liver tissue was associated with increased lipid peroxidation, with hepatic MDA levels approximately 420% higher. This was associated with reductions in hepatic GSH and TAC of about 61% and 59%, respectively. Likewise, diclofenac substantially increased renal MDA levels and renal GSH by about 262% each, while renal TAC decreased by approximately 57%. In the stomach, diclofenac increased MDA levels by approximately 208%, whereas GSH and TAC decreased by approximately 63% and 57%, respectively (Figure 2).

Relative to the diclofenac group, administration of oridonin 10 mg/kg substantially attenuated hepatic MDA levels by approximately 38%; meanwhile, hepatic GSH and TAC increased by 36% and 62%, respectively. In renal tissue, oridonin 10 mg/kg resulted in an approximate 46% decrease in MDA and a 46% decrease in GSH relative to diclofenac, while TAC increased by 42%. In gastric tissue, oridonin 10 mg/kg reduced MDA by about 29%, and raised GSH and TAC by 62% and 28%, respectively (Figure 2).

On comparison with the diclofenac group, oridonin 20 mg/kg had even stronger antioxidant effects. Hepatic MDA decreased by ∼ 63% with increased hepatic GSH and TAC by ∼101% and 141%, respectively. In the kidney, oridonin 20 mg/kg decreased MDA by ∼52%, decreased GSH by ∼61% relative to diclofenac, and increased TAC by ∼100%. MDA levels in gastric tissue decreased by about 49%, while GSH and TAC increased by about 131% and 84%, respectively (Figure 2).

Comparison of both doses of oridonin showed that oridonin 20 mg/kg was significantly more effective than oridonin 10 mg/kg in reducing MDA levels and improving antioxidant defenses in the liver, kidney, and stomach, providing evidence of a dose-dependent antioxidant effect (Figure 2).

### 3.4 Influence of oridonin on IRE1/TXNIP/Caspase-1 signaling pathways in liver and kidney

**FIGURE 3:**
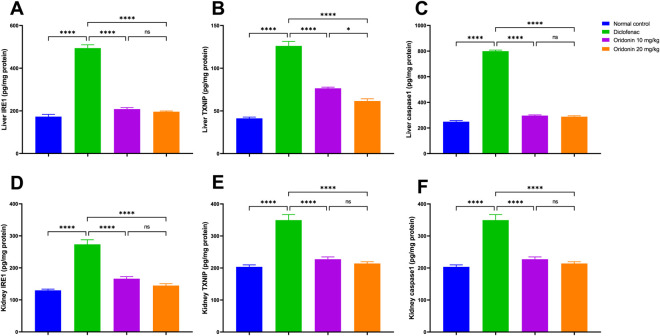
Influence of oridonin on the alterations in IRE1/TXNIP/Caspase-1 signaling pathways induced by diclofenac in liver and kidney tissues. **(A)** IRE1 levels in hepatic tissue, **(B)** TXNIP levels in hepatic tissue, **(C)** caspase-1 levels in hepatic tissue, **(D)** IRE1 levels in renal tissue, **(E)** TXNIP levels in renal tissue, and **(F)** caspase-1 levels in renal tissue. IRE1: inositol-requiring enzyme 1, TXNIP: thioredoxin-interacting protein. Data are expressed as mean ± SD; n = 6. Rats received oridonin (10 mg/kg and 20 mg/kg) for 7 days by gastric gavage followed by diclofenac (100 mg/kg, single IP dose) on the 8th day; * denotes p-value <0.05, **** denotes p-value <0.0001. This was determined using one-way ANOVA followed by Tukey multiple comparison *post hoc* tests.

Injection of diclofenac significantly increased IRE1 expression in the liver and kidney compared to the normal control by about 186% and 111%, respectively. Likewise, with diclofenac administration, TXNIP was elevated by around 206% in hepatic and 167% in renal tissues compared with normal rats. The expression of caspase-1 was also markedly upregulated by approximately 221% in the liver and 72% in the kidney, relative to the normal control (Figure 3).

Relative to the diclofenac group, pretreatment with oridonin 10 mg/kg significantly attenuated ER stress and inflammasome activation in both organs. Hepatic and renal IRE1 levels were reduced by approximately 58% and 39%, respectively, relative to the diclofenac group. TXNIP expression decreased by approximately 39% in the liver and 44% in the kidney, relative to diclofenac-treated rats, while caspase-1 levels were reduced by approximately 63% in hepatic tissue and 35% in renal tissue, relative to the diclofenac group (Figure 3).

Pretreatment with oridonin 20 mg/kg produced comparable protective effects. Hepatic and renal IRE1 expression was reduced by approximately 60% and 47%, respectively, relative to the diclofenac group. TXNIP levels decreased by approximately 51% in both liver and kidney, relative to diclofenac-treated rats, while caspase-1 expression was reduced by approximately 64% in hepatic tissue and 39% in renal tissue, relative to the diclofenac group (Figure 3).

Direct comparison between oridonin 10 mg/kg and oridonin 20 mg/kg revealed no statistically significant differences in hepatic or renal IRE1 and caspase-1 levels, while there was only a significant reduction in hepatic TXNIP level in oridonin 20 mg/kg group compared to oridonin 10 mg/kg group (Figure 3).

### 3.5 Influence of oridonin on CHOP, PERK, and NLRP3 expressions in liver and kidney

**FIGURE 4:**
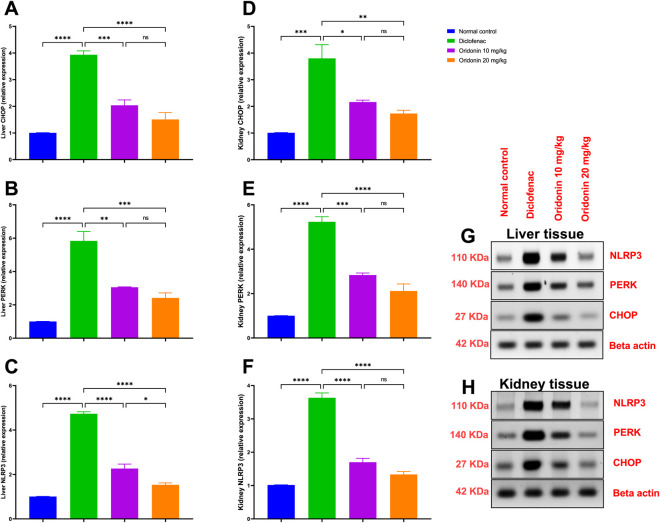
Influence of oridonin on the alterations in CHOP, PERK, and NLRP3 expression induced by diclofenac in liver and kidney tissues. **(A)** CHOP expression in liver, **(B)** PERK expression in liver, **(C)** NLRP3 expression in liver, **(D)** CHOP expression in kidney, **(E)** PERK expression in kidney, **(F)** NLRP3 expression in kidney, **(G)** Western blot images for liver, **(H)** Western blot images for kidney. NLRP3: NOD-like Receptor Family Pyrin Domain-Containing 3, PERK: Protein Kinase RNA-like Endoplasmic Reticulum Kinase, CHOP: C/EBP Homologous Protein. Data are expressed as mean ± SD; n = 6. Rats received oridonin (10 mg/kg and 20 mg/kg) for 7 days by gastric gavage followed by diclofenac (100 mg/kg, single IP dose) on 8th day; * denotes p-value <0.05, ** denotes p-value <0.01, *** denotes p-value <0.001, **** denotes p-value <0.0001; This was determined using one-way ANOVA followed by Tukey multiple comparison *post hoc* tests.

In the liver, diclofenac administration significantly increased CHOP, PERK, and NLRP3 expressions by approximately 293%, 483%, and 368%, respectively, relative to the normal control. Similarly, in the renal tissue, CHOP, PERK, and NLRP3 expression levels were significantly elevated by approximately 276%, 423%, and 256%, respectively, compared with normal controls (Figure 4).

Upon administration of oridonin 10 mg/kg, hepatic CHOP, PERK, and NLRP3 expressions were reduced by approximately 48%, 48%, and 52%, respectively, relative to diclofenac-injected rats. On the same line, oridonin 10 mg/kg reduced renal CHOP, PERK, and NLRP3 expressions by approximately 43%, 46%, and 53%, respectively, compared with the diclofenac group (Figure 4).

Furthermore, pretreatment with oridonin 20 mg/kg revealed a significant decline in hepatic CHOP, PERK, and NLRP3 expressions by approximately 62%, 59%, and 68%, respectively, relative to the diclofenac group. In the kidney, oridonin 20 mg/kg reduced CHOP, PERK, and NLRP3 expressions by approximately 54%, 59%, and 63% compared with diclofenac-injected rats (Figure 4).

Direct comparison between oridonin 10 mg/kg and Oridonin 20 mg/kg revealed no statistically significant differences in hepatic or renal CHOP and PERK expression. However, oridonin 20 mg/kg demonstrated a significant reduction in hepatic NLRP3 expression compared with oridonin 10 mg/kg (Figure 4).

### 3.6 Influence of oridonin on gastric tight junction proteins

**FIGURE 5:**
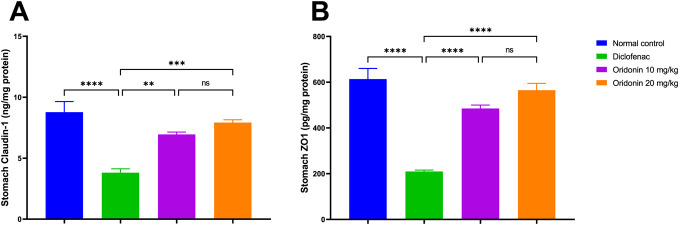
Influence of oridonin on the alterations in the gastric tight junction proteins induced by diclofenac. **(A)** Claudin-1 and **(B)** Zonula Occluden-1 (ZO-1). Data are expressed as mean ± SD; n = 6. Rats received oridonin (10 mg/kg and 20 mg/kg) for 7 days by gastric gavage followed by diclofenac (100 mg/kg, single IP dose) on the 8^th^ day; ** denotes p-value <0.01, *** denotes p-value <0.001, **** denotes p-value <0.0001; This was determined using one-way ANOVA followed by Tukey multiple comparison *post hoc* tests.

As observed in the diclofenac group, the gastric epithelial barrier integrity was significantly impaired compared with the normal control group. Claudin protein levels in the diclofenac group markedly decreased by approximately 56% in comparison with the normal control. Likewise, ZO-1 protein levels were around 66% lower compared to normal controls (Figure 5).

In comparison to the diclofenac group, tight junction protein expressions within the oridonin 10 mg/kg group were significantly increased. Claudin and ZO-1 levels were significantly elevated by around 82% and 131%, respectively, relative to diclofenac-administered rats (Figure 5).

The protective effect was more significant with oridonin 20 mg/kg treatment. Claudin and ZO-1 expressions were increased by approximately 108% and 169% compared to the diclofenac group. Claudin or ZO-1 protein levels did not significantly differ between oridonin 10 mg/kg and oridonin 20 mg/kg, suggesting a similar effect of both doses on maintenance of the gastric epithelial barrier with respect to protection (Figure 5).

### 3.7 Influence of oridonin on hepatic and renal NF-κB and IL-1β, and gastric occludin expressions

**FIGURE 6:**
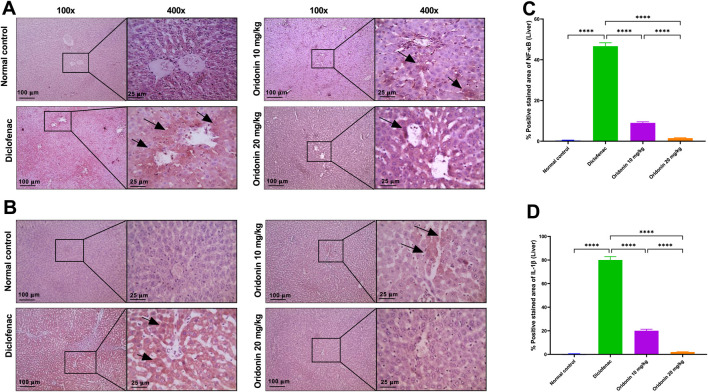
Influence of oridonin on the alterations in hepatic NF-κB and IL-1β expression induced by diclofenac. **(A,B)** Representative microscopic images of liver sections immunostained for **(A)** NF-κB and (B) IL-1β. **(C,D)** Histograms illustrating the percentage of positively stained areas for **(C)** NF-κB and **(D)** IL-1β expression. Normal control group showing negative staining, diclofenac group displaying strong positive brown staining in many hepatocytes (black arrows). Oridonin 10 and 20 mg/kg groups showing weak positive staining and completely negative IL-1β staining in 20 mg/kg group. IHC was counterstained with Mayer’s hematoxylin; magnifications are at ×100 (100 μm bar) and 400X (25 μm bar). Data are expressed as mean ± SD; n = 6. Rats received oridonin (10 mg/kg and 20 mg/kg) for 7 days by gastric gavage followed by diclofenac (100 mg/kg, single IP dose) on the 8th day; **** denotes p-value <0.0001. This was determined using one-way ANOVA followed by Tukey’s multiple-comparison *post hoc* tests.

**FIGURE 7:**
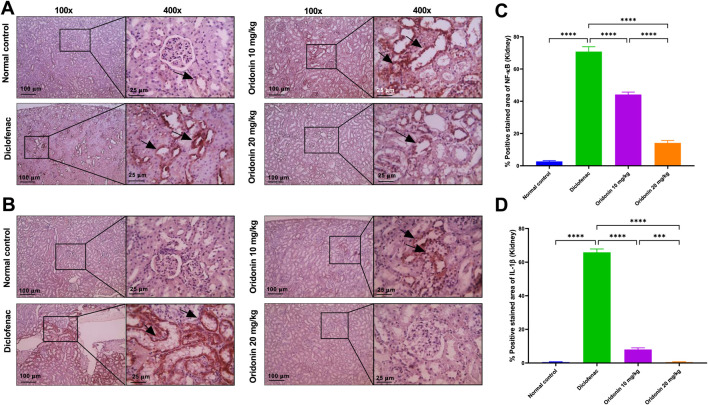
Influence of oridonin on the alterations in renal NF-κB and IL-1β expression induced by diclofenac. **(A,B)** Representative microscopic images of kidney sections immunostained for **(A)** NF-κB and **(B)** IL-1β. **(C,D)** Histograms quantify the percentage of positively stained areas for **(C)** NF-κB and **(D)** IL-1β. Normal control group showing very mild (NF-κB) or no (IL-1β) staining, diclofenac group displaying strong positive brown staining in many tubules (black arrows). Oridonin 10 and 20 mg/kg groups showing mild staining and completely negative IL-1β staining in 20 mg/kg group. IHC was counterstained with Mayer’s hematoxylin; magnifications are at ×100 (100 μm bar) and 400X (25 μm bar). Data are expressed as mean ± SD; n = 6. Rats received oridonin (10 mg/kg and 20 mg/kg) for 7 days by gastric gavage followed by diclofenac (100 mg/kg, single IP dose) on 8th day; *** denotes p-value <0.001, **** denotes p-value <0.0001; This was determined using one-way ANOVA followed by Tukey multiple comparison *post hoc* tests.

**FIGURE 8:**
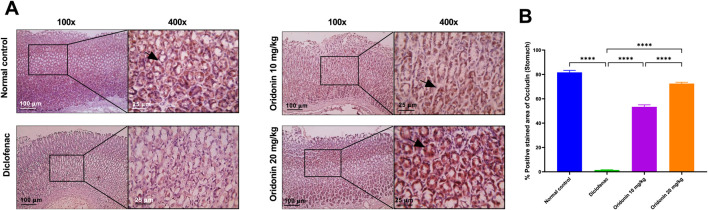
Influence of oridonin on the alterations in stomach occludin expression induced by diclofenac. **(A)** Representative microscopic images of glandular stomach sections immunostained for occludin. **(B)** Histogram quantifies the percentage of the stained area for occludin expression. Normal control group showing strong positive brown staining in many glandular cells (black arrows), Diclofenac group revealing weak positive staining. Oridonin 10 mg/kg group showing moderate positive staining, and Oridonin 20 mg/kg group showing strong positive staining. IHC was counterstained with Mayer’s hematoxylin; magnifications are at ×100 (100 μm bar) and 400X (25 μm bar). Data are expressed as mean ± SD; n = 6. Rats received oridonin (10 mg/kg and 20 mg/kg) for 7 days by gastric gavage followed by diclofenac (100 mg/kg, single IP dose) on 8^th^ day; **** denotes p-value <0.0001; This was determined using one-way ANOVA followed by Tukey multiple comparison *post hoc* tests.

In hepatic tissue, diclofenac administration resulted in a significant induction of NF-κB and IL-1β immunoreactivity by about 140 and 240-fold, respectively, compared with the normal control group. Similarly, in renal tissue, NF-κB and IL-1β expression were significantly elevated in the diclofenac group by about 27 and 132-fold, respectively, compared with controls. In the stomach, diclofenac reduced occludin expression by approximately 98% compared with the normal control group (Figures 6–8).

Relative to the diclofenac group, treatment with oridonin 10 mg/kg significantly attenuated inflammatory marker expression in both liver and kidney tissues. Hepatic NF-κB and IL-1β immunoreactivity were reduced by approximately 81% and 75%, respectively, compared with diclofenac-treated animals. In the kidney, Oridonin 10 mg/kg reduced NF-κB expression by approximately 38% and IL-1β expression by approximately 88% relative to the diclofenac group. In gastric tissue, Oridonin 10 mg/kg significantly increased occludin expression by approximately 36 folds compared with the diclofenac group (Figures 6–8).

Treatment with Oridonin 20 mg/kg produced more pronounced protective effects. Hepatic NF-κB and IL-1β expression were reduced by approximately 97% and 97.5%, respectively, relative to the diclofenac group (Figure 6). In renal tissue, Oridonin 20 mg/kg reduced NF-κB expression by approximately 80% and IL-1β expression by approximately 99% compared with diclofenac-treated animals (Figure 7). In stomach tissue, occludin expression increased by approximately 48 folds relative to the diclofenac group (Figures 6–8).

Direct comparison between Oridonin 10 mg/kg and Oridonin 20 mg/kg revealed significantly greater suppression of NF-κB and IL-1β expression in both liver and kidney tissues with the higher dose (Figures 6, 7). In gastric tissue, Oridonin 20 mg/kg produced a significantly greater restoration of occludin expression compared with Oridonin 10 mg/kg (Figure 8), indicating superior efficacy of the higher dose in preserving epithelial barrier integrity.

### 3.8 Influence of oridonin on histopathological changes

**FIGURE 9:**
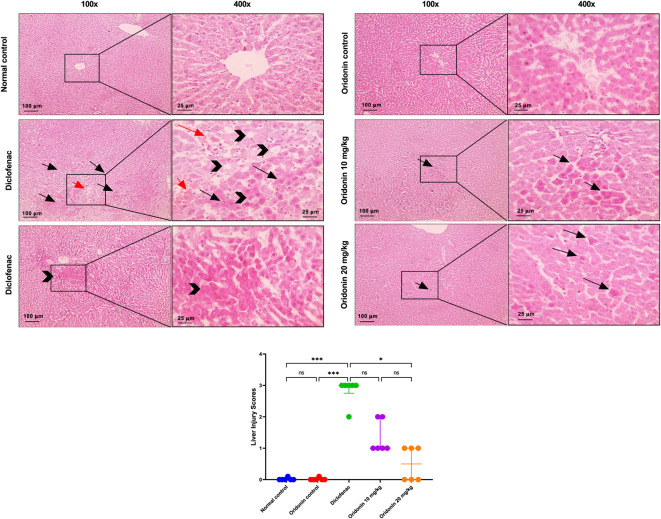
Influence of oridonin on the histopathological alterations induced by diclofenac in liver tissues. Microscopic pictures of H&E-stained rats’ liver in normal control and oridonin control group showing normal hepatocytes, central veins, portal areas and sinusoids. Diclofenac group showing many shrunken apoptotic hepatocytes with more eosinophilic cytoplasm (thin black arrow), few cytoplasmic vacuoles in other hepatocytes (black arrowhead), besides, congested central vein (red arrows). Other liver sections from diclofenac group showing focal area of coagulative necrosis (thick black arrow) with nuclear pyknosis and karyolysis. Oridonin 10 mg/kg group showing fewer shrunken apoptotic hepatocytes with more eosinophilic cytoplasm (thin black arrow). Oridonin 20 mg/kg group showing scattered few shrunken apoptotic hepatocytes (thin black arrow). Images are shown at ×100 magnification (100 μm scale bar) and ×400 magnification (25 μm scale bar). Scatter dot plots displaying liver injury scores. Data are expressed as median and IQR; n = 6. Rats received oridonin (10 mg/kg and 20 mg/kg) for 7 days by gastric gavage, followed by diclofenac (100 mg/kg, single IP dose) on the 8^th^ day; * denotes p-value <0.05, *** denotes p-value <0.001; This was determined using Kruskal–Wallis’ test with all pairwise comparisons via Dunn’s Method.

Figure 9 displays normal hepatocytes, central veins, portal areas, and sinusoids in the normal control and oridonin control groups. Hepatic specimens from rats in the diclofenac group show many shrunken, apoptotic hepatocytes with more eosinophilic cytoplasm, a few hepatocytes with cytoplasmic vacuoles, and a congested central vein. Other hepatic specimens from the diclofenac group reveal a focal area of coagulative necrosis with nuclear pyknosis and karyolysis. Hepatic specimens from rats in the oridonin 10 mg/kg group exhibit fewer shrunken, apoptotic hepatocytes with more eosinophilic cytoplasm. Additionally, hepatic specimens from the oridonin 20 mg/kg group show a few scattered shrunken, apoptotic hepatocytes.

**FIGURE 10:**
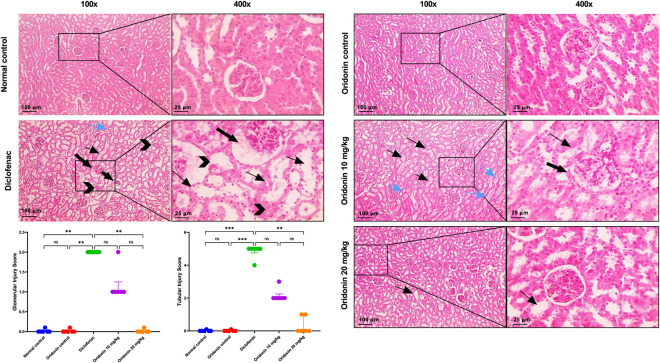
Influence of oridonin on the histopathological alterations induced by diclofenac in kidney tissues. Microscopic pictures of H&E-stained rats’ kidneys in normal control and oridonin control groups showing normal glomeruli, tubules and interstitial tissue. Diclofenac group showing diffuse severe tubular dilation with epithelial hydropic degeneration (thin black arrow), coagulative necrosis (black arrowhead) in tubular epithelium, cast formation (blue arrows), besides, shrunken glomerular tuft and markedly dilated Bowman’s space (thick black arrow) with eosinophilic material. Oridonin 10 mg/kg group showing decreased severity of tubular dilation (thin black arrow), cast formation (blue arrows), with little amount of eosinophilic material Bowman’s space (thick black arrow). Oridonin 20 mg/kg group showing mild tubular dilation (thin black arrow). Images are shown at ×100 magnification (100 μm scale bar) and ×400 magnification (25 μm scale bar). Scatter dot plots displaying glomerular and tubular injury scores. Data are expressed as median and interquartile range (IQR); n = 6. Rats received oridonin (10 mg/kg and 20 mg/kg) for 7 days by gastric gavage followed by diclofenac (100 mg/kg, single IP dose) on the 8th day; ** denotes p-value <0.01,*** denotes p-value <0.001, This was determined using Kruskal–Wallis’ test with all pairwise comparisons via Dunn’s Method.

Figure 10 shows normal glomeruli, tubules, and interstitial tissue in the control groups, including the normal control and the oridonin control. Renal specimens from the diclofenac group display widespread severe tubular dilation with epithelial hydropic degeneration, coagulative necrosis in the tubular epithelium, cast formation, as well as shrunken glomerular tufts and markedly dilated Bowman’s space containing eosinophilic material. Renal specimens from the oridonin 10 mg/kg group show reduced severity of tubular dilation and cast formation, with a small amount of eosinophilic material in Bowman’s space. Those from the oridonin 20 mg/kg group exhibited mild tubular dilation.

**FIGURE 11:**
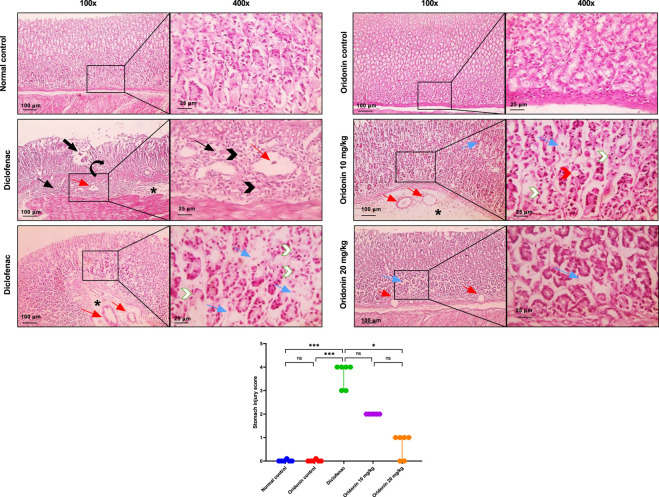
Influence of oridonin on the histopathological alterations induced by diclofenac in stomach tissues. Microscopic pictures of H&E-stained rats’ stomach in normal control and oridonin control groups showing normal glandular gastric mucosa and submucosa. Diclofenac group showing mucosal coagulative necrosis (curved black arrow) and ulceration (thick black arrow) associated with marked submucosal fibrosis (thin black arrow) infiltrated by leukocytic cells (black arrowheads), besides, edema (*) and dilated blood vessels (red arrows). Moreover, diclofenac group showing shrunken necrotic glandular structures (white arrowheads) with interstitial edema (blue arrow) associated with marked submucosal edema (*) and dilated blood vessels (red arrows). Oridonin 10 mg/kg group showing vacuolar degeneration in glandular epithelium (red arrowheads), shrunken necrotic glandular structures (white arrowheads) with interstitial edema (blue arrow) in mucosa with marked submucosal edema (*) and dilated blood vessels (red arrows). Oridonin 20 mg/kg group showing decreased interstitial edema (blue arrow) with mildly dilated blood vessels (red arrows) in mucosa. Images are magnified at ×100 (with a 100 μm scale bar) and 400X (with a 25 μm scale bar). Scatter dot plots displaying stomach injury scores. Data are expressed as median and interquartile range (IQR); n = 6. Diclofenac (100 mg/kg, single IP dose); Oridonin (10 or 20 mg/kg, oral for 7 days). * denotes p-value <0.05, *** denotes p-value <0.001; This was determined using Kruskal–Wallis’ test with all pairwise comparisons via Dunn’s Method.

Figure 11 shows normal glandular gastric mucosa and submucosa in the control groups, including the normal control and oridonin control groups. The glandular stomach from the diclofenac group displays mucosal coagulative necrosis and ulceration, accompanied by significant submucosal fibrosis infiltrated by leukocytic cells, as well as edema and dilated blood vessels. Other gastric sections from the induction group reveal shrunken, necrotic glandular structures with interstitial edema, along with prominent submucosal edema and dilated blood vessels. The glandular stomach from the oridonin 10 mg/kg group shows vacuolar degeneration of the glandular epithelium, shrunken, necrotic glandular structures, interstitial edema in the mucosa, and marked submucosal edema with dilated blood vessels. The glandular stomach from the oridonin 20 mg/kg group exhibits decreased interstitial edema and mildly dilated blood vessels in the mucosa.

Comparison between the injury scores in normal control group and the diclofenac group demonstrated marked histopathological damage across all examined organs. Diclofenac administration resulted in significant increases in hepatic, renal, and gastric injury scores compared with the normal control group (Figures 9–11).

Comparison of the oridonin control group with the normal control group revealed no statistically significant differences in glomerular, tubular, hepatic, or gastric histological injury scores, indicating that oridonin alone did not induce tissue injury (Figures 9–11).

In oridonin 10 mg/kg group, renal, hepatic, and gastric architecture improved, while residual injury was apparent. Greater improvement was observed with oridonin at 20 mg/kg. Glomerular injury was absent, and tubular, hepatic, and gastric injury scores were significantly decreased compared with the diclofenac group (Figures 9–11).

## 4 Discussion

The current study provides further evidence of the robust protection provided by oridonin against diclofenac-induced multi-organ toxicity in rats with respect to nephrotoxicity, hepatotoxicity, and gastric injury. With the protection with oridonin, biochemical indices of organ function, histopathologic architecture and molecular indices of oxidative stress and inflammation were significantly modulated. These results adhere to emerging literature on the multi-organ protection of oridonin and associated phytochemicals in drug-induced organ injury models (Khalil et al., 2025; Oyinloye et al., 2023; Varışlı et al., 2023).

Diclofenac, a common NSAID, is reported to cause dose-dependent toxicity in hepatic, kidney, and gastrointestinal systems predominantly via oxidative stress, mitochondrial failure, and inflammation-driven pathways (Kei and Uesawa, 2025; Kim et al., 2024; Mraisel et al., 2024; Priya et al., 2025). In this experiment, rats injected with diclofenac showed increased serum markers of hepatic (ALT and AST) and renal (BUN and creatinine) injury, increased lipid peroxidation, and histological evidence of hepatocellular necrosis, tubular degeneration, and erosion of gastric mucosa. Oridonin co-administration remarkably reversed these changes with reconstitution of antioxidant enzyme activities (GSH and TAC), reduction of MDA, and preservation of tissue architecture.

Crucially, oridonin pretreatment affected the central molecular pathways involved in diclofenac toxicity, the latter primarily through inhibition of the TXNIP/NLRP3 inflammasome pathway, reduction of ER stress markers, and maintenance of tight junction protein expression in the gastric mucosa. Taken together, these findings indicate that the action of oridonin is multivalent in reducing the adverse influence of diclofenac (He et al., 2018; Huang et al., 2024; Zhao et al., 2023).

Diclofenac-induced organ injury has complex pathogenesis which includes a variety of direct and indirect pathways. Diclofenac’s treatment and toxic effects are mediated predominantly by the inhibition of COX enzymes, resulting in reduced prostaglandin synthesis. This underlies the anti-inflammatory efficacy, but it impairs physiological prostaglandin-mediated cytoprotection of the liver, kidneys, and gastrointestinal tract (Kei and Uesawa, 2025; Mraisel et al., 2024; Priya et al., 2025).

Nephrotoxicity is due to decreased renal blood flow following inhibition of prostaglandin, resulting in ischemia, tubular atrophy, and necrosis. Diclofenac also enhances oxidative stress in renal tissues, with upregulation in ROS production and lipid peroxidation, in addition to depletion of endogenous antioxidants like GSH (Alkuraishy et al., 2019; Mraisel et al., 2024). Renal damage is further exacerbated by inflammatory cytokines (e.g., TNF-α and IL-1β) and NLRP3 inflammasome activation (Alkuraishy et al., 2019; Zhang et al., 2022).

Mitochondrial dysfunction and oxidative stress are the major contributors to hepatotoxicity. Diclofenac and its reactive metabolites impair mitochondrial oxidative phosphorylation, block electron transport chain complexes, and drive the opening of the mitochondrial permeability transition pore to deplete ATP, release cytochrome c, and activate the caspase cascade, ultimately triggering apoptosis (Kim et al., 2024; Parthasarathy and Evan Prince, 2021; Priya et al., 2025; Varışlı et al., 2023). Acyl glucuronide metabolites are produced, and covalent binding to cellular macromolecules exacerbates immune-mediated and idiosyncratic liver injury (Priya et al., 2025).

NSAID-induced gastric injury is closely associated with disruption of epithelial tight junctions and increased mucosal permeability. Diclofenac substantially decreased expression of claudin, ZO-1, and occludin, confirming impairment of gastric barrier integrity as shown in the present study. Oridonin effectively restored these tight junction proteins, suggesting the maintenance of epithelial cohesion and barrier function. This is an important finding as direct evidence that oridonin played a protective role against NSAID-induced gastric injury has proved to be scarce. The restoration of tight junction proteins could imply that oridonin not only reduces inflammation and oxidative stress but also promotes epithelial repair mechanisms.

A comprehensive understanding of the cross talk among the following pathways is essential to strengthening the mechanistic interpretation of the study findings. Oxidative stress, endoplasmic reticulum (ER) stress, and NLRP3 inflammasome activation represent a tightly interconnected signaling network that plays collectively a central role in the cellular injury propagation. Excessive generation and accumulation of ROS acts as an initiating factor that disrupts redox homeostasis and induces protein misfolding, thereby trigger ER stress and activate unfolded protein response pathways, including PERK, IRE1, and CHOP signaling cascades (Li et al., 2020). Sustained ER stress further aggravates oxidative damage through calcium dysregulation and mitochondrial dysfunction, forming a positive seif-amplifying feedback loop (Li et al., 2020; De Deus et al., 2023). Interestingly, both oxidative and ER stress converge on the stimulation of the NLRP3 inflammasome, primarily via upregulation of TXNIP, which dissociates from thioredoxin under oxidative conditions and directly bind to NLRP3 to promote inflammasome (De Deus et al., 2023). This activation promotes caspase-1 cleavage and the release of pro-inflammatory cytokines, including IL-1β and IL-18 (Huang et al., 2024; Luo et al., 2024; Sbai et al., 2024). Moreover, inflammasome activation can further enhance mitochondrial ROS production, reinforcing a vicious cycle of oxidative stress and inflammation (De Deus et al., 2023). Collectively, these pathways operate in a coordinated and self-amplifying manner, ultimately driving inflammatory responses and contributing to induction and progression of multi-organ damage.

Diclofenac toxicity is characterized by enhanced ROS production accompanied by oxidative stress through depletion of antioxidant defenses. The oxidative milieu is damaging to cellular macromolecules and is a harbinger of downstream inflammatory and apoptotic pathways (Alkuraishy et al., 2019; Kibibi Wairimu, 2025; Kim et al., 2024; Parthasarathy and Evan Prince, 2021). The major role of the TXNIP/NLRP3 inflammasome axis in NSAID-induced organ injury has recently been revealed with high importance. Under oxidative stress, TXNIP gets liberated from thioredoxin and interacts with NLRP3 to enhance the assembly of inflammasomes, activation of caspase-1, and maturation of pro-inflammatory cytokines IL-1β and IL-18 (Huang et al., 2024; Luo et al., 2024; Sbai et al., 2024; Zhang et al., 2022).

A significant strength of this study is the comprehensive characterization of ER stress and inflammasome-induced pathways. Diclofenac significantly stimulated ER stress sensors (such as IRE1, PERK, and CHOP) in hepatic and renal tissues. Prolonged ER injury also drives apoptotic and inflammatory cell death contributing to further organ damage. Oridonin markedly suppressed these markers, suggesting an effective removal of ER stress burden.

ER stress, through the unfolded protein response (UPR), additionally amplifies TXNIP and NLRP3 expression, interacting oxidative, inflammatory and apoptotic pathways, forming a reciprocal loop (Ong et al., 2024; Zhang et al., 2022). ER stress is being acknowledged as an important mediator of drug induced organ toxicity. Diclofenac can upregulate ER stress markers (ATF6, PERK, IRE1, GRP78), disturb the protein folding system, and induce apoptotic signaling via CHOP and caspase activation (Ong et al., 2024; Repges et al., 2023; Varışlı et al., 2023; Zhou et al., 2023). The crosstalk between ER stress and inflammasome hyperactivation was established through TXNIP as a core mechanism in multi-organ damage mediated by NSAIDs (Ong et al., 2024; Zhang et al., 2022). At the same time diclofenac activated the TXNIP/NLRP3/caspase-1 axis, which is a significant pathway from oxidative stress to inflammasome-driven pyroptosis.

In this study, Increased expression of TXNIP and NLRP3 in association with elevated expression levels of caspase-1 and IL-1β immunoreactivity supported inflammasome activation in liver as well as renal tissues. Oridonin significantly attenuated these responses, indicating its strong inhibitory role of sterile inflammation. This inhibition probably is due to upstream antioxidant actions as well as direct hindrance of inflammasome assembly, as described previously for oridonin.

Histopathological examination confirmed the biochemical and molecular findings, finding widespread hepatocellular degeneration, renal tubular injury, and gastric mucosal disruption after diclofenac administration. Oridonin markedly improved tissue architecture, reducing apoptotic features and inflammatory infiltration. Immunohistochemical studies additionally clarified the inhibition of NF-κB and IL-1β signaling in liver and kidney tissues, further supporting the claim that oridonin significantly inhibits inflammatory amplification at the tissue level.

The protective properties of oridonin reported in this study are supported by a growing body of literature. Oridonin showed hepatoprotective, nephroprotective, and gastroprotective effects in various models of drug-induced and inflammatory organ injury (Huang et al., 2024; Zhang et al., 2025; Zhao et al., 2023; Zhou et al., 2023; Zhu et al., 2025). In models of LPS/D-galactosamine-induced acute liver injury, oridonin enhanced survival, decreased levels of aminotransferase, and inhibited pro-apoptotic signaling. In ischemia-reperfusion and diabetic nephropathy models, oridonin reduced renal dysfunction, inflammatory cytokine production, and inhibited TLR4/NF-κB and p38-MAPK pathways (Huang et al., 2024).

Oridonin’s ability to inhibit the TXNIP/NLRP3 inflammasome is proven in many studies. He et al. (2018) reported that oridonin covalently binds to NLRP3 to inhibit its activation and downstream inflammatory responses (He et al., 2018). Other reports have also shown that oridonin is effective against inflammasome activation and organ damage in models of colitis, acute lung injury, and diabetic nephropathy (Huang et al., 2024; Zhu et al., 2025). Properties that protect the barrier have also been shown to be provided by oridonin in intestinal, retinal, and gastric models. Tight junction protein levels are also enhanced by oridonin itself, and epithelium conservation is underlined by oridonin (Zhu et al., 2025). The latter observations are in agreement with the present study’s finding of oridonin-mediated retention of tight junctions between the gastric mucosa.

An important issue that should be highlighted, reports of hepatotoxicity in relation to high doses or prolonged administration in a few experimental models suggest that dose optimization and comprehensive safety evaluation are essential prior to therapeutic application. There are other considerations for drug–herb interactions. And, in particular, it has been shown that oridonin may affect CYP2C and CYP3A encoding enzymes in the body, which brings to light potential pharmacokinetic interactions with diclofenac and also other co-administered drugs that may use these CYP2C and CYP3A pathways. This consideration needs to be further pursued in the context of polypharmacy. Therefore, detailed toxicological assessments, dose–response investigations, and studies evaluating CYP-mediated metabolic interactions are essential to fully define its safety profile and support its clinical translation.

## 5 Conclusion

The current study has thus far elucidated the significant protective efficacy of oridonin against diclofenac-mediated multi-organ toxicity involving the kidney, liver, and stomach. Diclofenac administration resulted in significant functional impairment, oxidative stress, inflammatory overactivity, ER stress induction, and epithelial barrier disruption. Oridonin pretreatment effectively counteracted these pathological changes, as evidenced by the normalization of renal and hepatic biomarkers, restoration of antioxidant defenses, repression of ER stress and inflammasome-related signaling, and retention of gastric tight junction proteins. The synergistic modulation of oxidative, inflammatory responses, and stress-response pathways exemplify the pleiotropic nature of oridonin’s cytoprotective effects.

Together, these results confirm that oridonin disrupts the cascades between the interrelated molecular pathways involved in NSAID-driven organ injury and emphasize its usefulness as a multi-target defense agent of diclofenac-induced toxicity. It is suggested that further studies be conducted to explore the therapeutic application and potential of oridonin to develop the findings of this study. It should be investigated in future studies to ascertain its effects in chronic diclofenac exposure to model human diclofenac exposure, consistent with continued clinical use of NSAID dosing.

## 6 Limitations of the study

There are some limitations that must be noted. While the rat model is informative, trans-species differences in diclofenac metabolism (e.g., cytochrome P450 isoform expression and glucuronidation capacity) that introduce translational problems. To improve translational relevance, future studies should incorporate the use of human-relevant models to improve translational accuracy. For example, precision-cut tissue slices maintain the native organ-specific architecture, cellular diversity, and metabolic enzyme activity of human organs, enabling more physiologically relevant evaluation of drug metabolism and toxicity. In parallel, advanced three-dimensional (3D) cell culture models, such as human organoids and spheroids, better mimic the structural and functional complexity of human tissues compared to traditional mono layer cultures. These models can provide more accurate insights into human-specific responses, including drug-induced injury and protective mechanisms. Integrating such models with preclinical animal data would strengthen the predictive value of future studies and facilitate the translation of oridonin as a potential therapeutic strategy for diclofenac-induced organ toxicity. Additionally, the lack of clinical data is yet another notable constraint. These findings add to a critical yet currently unrecorded body of preclinical evidence supporting whether or not oridonin is effective and safe for NSAID-induced organ injury. While there is the potential for data on the efficacy and safety of oridonin via ongoing clinical studies of the drug for other indications, studies to confirm its efficacy for this condition will be necessary.

## Data Availability

The original contributions presented in the study are included in the article/supplementary material, further inquiries can be directed to the corresponding author.
